# Drawing to Remember: External Support of Older Adults’ Eyewitness Performance

**DOI:** 10.1371/journal.pone.0069937

**Published:** 2013-07-29

**Authors:** Coral J. Dando

**Affiliations:** Department of Psychology, University of Wolverhampton, Wolverhampton, United Kingdom; University College London, United Kingdom

## Abstract

Although healthy aging is accompanied by a general decline in memory functioning, environmental support at retrieval can improve older adults’ (+65 years) episodic remembering. Despite those over the age of 65years representing a growing proportion of the population, few environmental retrieval support methods have been empirically evaluated for use with older witnesses and victims of crime. Here, the efficacy of a novel retrieval technique, the Sketch Mental Reinstatement of Context, is compared with a standard Mental Reinstatement of Context and a no support control (Control). Fifty-one participants witnessed an unexpected live event, and 48 hours later were interviewed using one of three aforementioned techniques. In line with predictions emanating from cognitive theories of aging and the environmental support hypothesis, participants in the Sketch Mental Reinstatement of Context condition recalled significantly more correct information and fewer inaccurate items. The Sketch Mental Reinstatement of Context technique appears to scaffold memory retrieval in an age-appropriate manner during a post-event interview, possibly by encouraging more effortful retrieval and reducing dual-task load. As such, this procedure offers an effective alternative to current approaches, adding to the toolbox of techniques available to forensic and other interviewers.

## Introduction

Healthy aging is accompanied by a decline in memory performance. However, age-related deficits affect some *types* of memory more than others. Episodic retrieval, the ability to remember our own experiences in a different temporal and spatial context to that of encoding [1], is particularly vulnerable ([2], [3]), and reductions in episodic performance are especially pronounced in free- and cued-recall tasks ([4], [5]). Yet, within many criminal justice systems, older witnesses and victims are required to recount episodic experiences during interview procedures that largely comprise free- and cued-retrieval tasks ([6], [7]).

Those over the age of 65 years represent a sizable and growing proportion of the witness population, who with greater mobility and financial independence are also increasingly the victims of certain types of crime, such as distraction burglary [8], financial crimes [9], elder abuse, and neglect [10]. Yet, the literature investigating older eyewitness memory performance in interviews is sparse, and there exist few rigorously tested, theoretically supported tools for assisting this particular subset of the population to recall an experienced event. Rather, current methods for collecting episodic eyewitness information are driven by techniques that are performance contra indicators for older adults.

Enhancing older adults’ access to justice by supporting their episodic remembering is timely, but doing so presents significant challenges. Eyewitness cognition is complex, because encoding environments are typically less than optimal. Moreover, eyewitness memory is highly malleable ([11], [12]). Hence, those who seek to develop practical procedures for eliciting eyewitness information must be cognizant of the need to control and manage the retrieval environment to ameliorate post event contamination. The Environmental Support Hypothesis offers one framework upon which to develop an age appropriate eyewitness interview technique, it having been found that older adults’ episodic remembering can be improved if their cognitive processes are supported and the retrieval task is managed so as to reduce situational demands ([13], [14], [15]). Environmental retrieval strategies found to be beneficial for older adults include external memory aids, such as notes, the provision of appropriate retrieval cues, and the promotion of slow-accurate strategies ([16]). With reference to this literature, the research reported here investigates the efficacy of an environmental support method that promotes externalization with a view to increasing available processing resources, so assisting older adults’ eyewitness remembering in a domain where errors can have real and lasting consequences.

The Cognitive Interview technique (CI) [17] is the prevalent empirically-informed technique for retrieving episodic information from all cooperative witnesses and victims. Designed to reduce errors of omission (forgetting) and commission (confabulations) without a concomitant increase in intrusions (reporting of inaccurate information), the CI is a homogeneous procedure comprising several distinct mnemonic components and retrieval support strategies [18]. One of the core CI components is the Mental Reinstatement of Context technique (MRC), which draws upon the encoding-specificity principle of memory [19]. Encoding specificity provides a general theoretical framework for understanding how contextual information affects memory. Specifically, that memory is improved when information available at encoding is also available at retrieval. Comprising a series of instructions, the MRC technique encourages witnesses to mentally recreate both the psychological and physical environment that existed at the time of the to-be-remembered (TBR) event in an attempt to facilitate the feature overlap between the event and the retrieval environment.

The beneficial effect of mentally reinstating the context is well established for eyewitness memory. Componential research indicates that the MRC technique is one of the most effective individual components of the CI procedure for both children and adults <65 years ([20]–[25]). However, the effects of MRC are known to vary [26], and little is known about the utility of the MRC technique with older adults. The suitability of the CI procedure *per se* (which includes the MRC technique) for older adults has received some empirical support, the procedure as a whole having been found to be an effective method of increasing correct remembering for this group. When interviewed using a CI, older mock witnesses tend to outperform those interviewed using a control interview technique, often recalling more correct information without a concomitant increase in errors ([27], [28], [29]).

However, the literature pertaining to older eyewitness performance is in its infancy, the number of published studies is small, and the control (comparison) interviews generally exclude any environmental support. Moreover, because there exists no componential investigation of the relative contribution of the individual CI techniques, their suitability for older witnesses is unknown. Currently, the literature merely supports the notion that, for this group, an interview procedure that includes some environmental support at retrieval improves episodic remembering compared with a similarly structured procedure that provides no support.

The environmental support hypothesis, and cognitive theories of aging, would predict that the current MRC procedure is unlikely to be the *most* effective method of facilitating the feature overlap between the experienced event and the retrieval environment for older adults. The current procedure comprises a series of verbal instructions, which are applied individually and incrementally over a period of time (see Appendix S1). Using in excess of 20 short instructions witnesses are instructed to close their eyes and listen carefully to what the interviewer says, and to silently reconstruct numerous images and mental states accordingly, before being ‘allowed’ to verbalise event information. This method demands that older adults complete a number of internal, concurrent hippocampus-dependent cognitive operations. For example, having to pay attention to, and understand the interviewer’s instructions at the same time as constructing and maintaining several mental images over time, all at a pace dictated by the interviewer. Yet older adults typically exhibit reduced processing resources [30] and deficits in working memory/executive control [31]. Hence, the demands associated with constructing and maintaining a mental image while receiving and understanding additional cues are likely to outstrip the cognitive resources available. Difficulties in associating single units of information ([32], [33], [34]), and reductions in attentional capacity ([35], [20]) are also well documented. Hence, it follows that older witnesses might be better served by a modified mental reinstatement of context retrieval technique designed to overcome these deficits.

Recent research conducted with adult mock witnesses between the ages of 18 and 39 years has shown a Sketch Mental Reinstatement of Context technique (Sketch MRC) to be an effective and efficient retrieval support tool. The technique was devised as a replacement for the current Mental Reinstatement of Context (MRC), specifically for use by less experienced, frontline police interviewers (who typically receive minimal interview training), to limit interviewer contamination and reduce the time taken to conduct volume crime witness interviews ([20], [22[, [22]). Participants interviewed using the Sketch MRC were found to perform equally to, or better than those in the current MRC condition for the amount of correct information with no increase in the reporting of inaccurate items (inaccurate information is discrepant from that which occurred in the stimulus, for example saying that the dog was black, rather than the dog was brown).

Cognitive control and speed of processing accounts indicate that age-related episodic retrieval deficits may emanate from a slowing of cognitive processes, reduced processing efficiency, and diminished working memory capacity ([36], [37], [38]). However, recall performance can be improved when uncomplicated environmental retrieval support is in place to (i) scaffold the psychological mechanisms by which people actively maintain information and instructions for short periods of time, and how they use this information to guide and control their behavior and (ii) when sufficient time is allowed to process cognitive tasks because increased age is associated with a decrease in the speed with which processing operations are completed. Hence, cognitive performance is reduced because early processing is no longer available when later processing is complete ([39], [40]). The Sketch MRC technique naturally allows such age-related adjustments. There are fewer instructions, and they are straightforward. Moreover, witnesses naturally dictate the pace of recall, ensuring sufficient time to think about and understand the instructions, which in turn may reduce the situational demands experienced by older adults.

Every witness’s experience is individual, and subjective [41]. Accordingly, the ‘one size fits all’ approach to mental reinstatement of context currently taught to police investigators may be inappropriate, for example leading to the provision of incompatible retrieval cues, which are known to impair episodic retrieval performance. Incompatible/inappropriate retrieval cues are particularly problematic for older witnesses for whom the negative effects of suboptimal retrieval cues are compounded ([42], [43]) because such cues degrade their ability to make meaningful connections between the to-be-remembered elements of an event ([32], [33]). An additional benefit may arise from encouraging witnesses to access their own contextual retrieval cues through Sketch MRC rather than relying on retrieval cues provided by the interviewer. Indeed, age differences are reduced in tasks that provide *efficient* cues at retrieval ([13], [44], [45]), that is, cues actually associated with the encoded event.

The current research investigated, for the first time, the efficacy of the Sketch MRC for helping older adult witnesses (>65years) to retrieve episodic information when being interviewed about a live, unexpected event. Already shown to be effective with adults <65 years, and offered as a method for assisting children (and other vulnerable populations) to reinstate the context [46], this research is timely. Older adults’ episodic performance is not compared with younger adults: the developmental literature in this domain is vast, and it is well documented that younger eyewitnesses’ episodic performance is typically superior to that of older eyewitnesses. Rather, this research concerns empirically evaluating a method for improving older adults’ eyewitness performance in applied settings. The contemporary theoretical and empirical eyewitness literature, and the environmental support hypothesis suggest that the Sketch MRC technique will be more effective for supporting older adults’ episodic retrieval than the currently advocated MRC procedure and no environmental support.

## Methods

This research was approved by the Lancaster University Research Ethics Committee, and all participants in this study provided written consent.

### Design

A between-subjects design was employed. Retrieval interview was the independent variable, with three levels: Mental Reinstatement of Context; Sketch Mental Reinstatement of Context; and Control (no support). Dependent variables were (i) the amount of correct, inaccurate and confabulated items of information recalled as a function of condition (global performance), (ii) the amount of correct, inaccurate and confabulated items of information recalled as a function of interview retrieval phase (phase performance). In addition, the type of information (action; objects; person) recalled was identified.

### Materials

Global cognitive status was determined using the Mini Mental States Examination [47] and the Geriatric Depression Scale [48]. The Mini Mental States Examination (MMSE), which screens for cognitive impairment without obscuring the effects of age on recall, was administered individually to each participant. This is a short test (about 10 minutes in duration) comprising 20 questions that assess orientation, attention, language abilities, immediate and short-term recall, as well as the ability to follow simple verbal commands [49]. No participant scored below 26 on this measure, indicating the absence of abnormal cognitive impairment. The Geriatric Depression Scale (GDS -15) is a 15-item questionnaire designed to screen for depressive symptoms in older adults. No participant scored over 5 on this measure, indicating the absence of abnormal depressive symptoms.

### Participants

Fifty-one adults participated in this research, 17 males and 34 females. The mean age of the participants was 69.9 years (*SD* 4.98 years) ranging from 67 to 89 years. All lived independently in the community, and were recruited directly via two community organizations that allowed the research team access to their mailing lists to invite members to a series of community presentations entitled ‘Introducing Psychology’. Participants were not aware that they would be asked to take part in a research study.

### Procedure

This research employed a ‘live’ mock witness event that took place partway through the presentation (after 20 minutes had elapsed), after which the speaker continued presenting for a further 25 minutes. Two actors, one male and one female entered the seminar room (a large room with seating for an audience of approximately 75, and with overhead projection facilities and a podium at the front), and approached and interrupted the speaker, who at the time was presenting to an invited audience of approximately 60 attendees including the study participants. A conversation ensued concerning whether or not the actors should be attending this lecture. There was a further brief verbal exchange between the speaker and the actors concerning room bookings and possible solutions to the problem, at which point the female actor used her cell phone to call a friend, while the male actor consulted his diary. Both the actors left the room, apologising for the confusion. The interruption lasted for one minute.

Once the presentation was complete, the researcher entered the seminar room, explained that what had occurred mid-way through was part of a research project. She then requested attendees to participate in the project, providing them with information sheets, answering questions, and obtaining signed consent forms. Participants were randomly allocated to one of the three interview conditions (Sketch MRC; MRC; Control) and then left after having made an appointment for the researcher to conduct a face-to-face interview 48 hours later (participants were naive to the interview conditions). It was explained to participants (by the experimenter, and on the information sheets that accompanied the consent forms) that during the interview they would be asked some questions about the presentation.

### Interviews

All of the interviews were similarly structured, comprising the following phases: (i) greet and explain, (ii) rapport, (iii) free recall, (iv) questioning, and (v) closure. They comprised the same number of retrieval attempts in the same order, and only differed in the Free Recall phase during which the experimental manipulation took place. One experienced interviewer conducted all of the interviews, following condition-appropriate protocols verbatim, which were based on the current UK investigative interview model ([7], [50]). In brief, the interview procedures were as follows (detailed interview protocols are available from the author):

All interviews commenced with a *greet and explain* phase, during which the interviewer greeted the participant, introduced herself, and explained what the interview would entail. In addition, each participant was given an opportunity to ask any questions, and permission was again sought for the interview to be audio recorded. The interviewer then moved seamlessly into the *rapport* phase, during which she interacted meaningfully with the participant, contributing as an interested party, using open-ended invitations to exchange information and to demonstrate an understanding of the situation from the participant’s point of view [51].

#### Sketch mental reinstatement of context interview

The *free recall* phase of interviews in this condition commenced with each participant being provided with paper and pencils, and then being asked to draw the to-be-remembered event in as much detail as possible, and to describe each item/event as they were drawing (See Appendix S2, also see [20]). Participants were instructed to draw anything they wished and whatever reminded them of the event. Participants were given unlimited time to draw, following which the interviewer instructed the participant to: (i) “please explain what you remember about the event you saw a few days ago”, (ii) “I only want you to tell me what you *actually* remember, please don’t guess”, (iii) “if you can’t remember just say so” (from hereon referred to as the Retrieval Instructions).

#### Mental reinstatement of context interview

The *free recall* phase of interviews in this condition commenced with interviewer giving instructions aimed at aiding the interviewee to mentally reinstate both the physical and psychological context that existed at the time of encoding in line with the procedure currently taught to police interviewers (see Appendix S1: [52], [50]). The instructions were delivered slowly and deliberately, and in between each instruction the interviewer paused for 10 seconds to allow enough time for the participant to reinstate the context as instructed. Following this participants were given the Retrieval Instructions.

#### Control interview

The *free recall* phase of interviews in this condition commenced with the interviewer giving the retrieval instructions with no further instruction.

The *questioning* phase of each interview immediately followed the *free recall* phase. Prior to the commencement of this phase, all participants were again given the retrieval instructions, following which the interviewer questioned each participant in a manner compatible with the way in which he/she had recalled the event during the free recall phase. To do this, the interviewer used the notes made during that free recall phase, asking one question about each of the topics recalled. Thereafter, the interviewer completed the *closure* phase, thanking the participant for his/her participation, and offering an opportunity to ask questions.

### Coding and Scoring

The live event was discretely digitally audio- and video-recorded and later used to construct a scoring and coding template (cf. [20], [21], [53]). A comprehensive list of events in the film was compiled, totalling 97 details: 46 person, 25 actions, and 26 objects. Using the coding template, each of the interviews was transcribed and scored for the number of information items verbalised from the commencement of the *free recall* phase until the end of the *questioning* phase that were correct, inaccurate (e.g., saying that the man’s bag was black, when in fact it was brown), and confabulated (mentioning a detail or event that was not present or did not happen). The phase within the interview that the information was verbalised was noted (free recall or questioning), and information items were only scored once (i.e., information was scored the first time it was mentioned, but disregarded if mentioned subsequently). Duration and number of questions asked by the interviewer were also noted. Drawings produced by the participants in the Sketch MRC were not coded.

Twenty-five interviews were selected at random and coded independently by a research assistant who was naive to the aims of the experiment and hypotheses. Analysis of inter-rater reliability revealed very good reliability for all three measures: total correct, Kappa = .791, *p* = .003; total inaccuracies, Kappa = .841, *p* = .003; total confabulations, Kappa = .921, *p*<.001.

## Results

### Manipulation Checks

No significant differences emerged across the conditions for age, MMSE scores, GDS scores, interview duration, and the number of questions asked, all *Fs* <1.134, all *p*s >.05 (see Table 1 for the manipulation means and standard deviations).

**Table 1:** Mean (SDs in parenthesis) age, MMSE, GDS, questions asked, and interview duration.

	Retrieval Condition		
	Sketch MRC	MRC	Control
Geriatric Depression	5.65 (4.04)	4.76 (3.66)	5.25 (3.01)
Mini Mental State	28.88 (1.01)	29.36 (0.81)	29.89 (1.07)
Age	70.70 (6.43)	69.80 (6.77)	71.60 (5.93)
Interview Duration	18.12 (8.43)	21.01 (7.02)	17.02 (5.12)
No of questions asked	13.41 (6.07)	11.04 (4.19)	10.41 (6.18)

### Analysis Approach

Eyewitness memorial performance is typically assessed by analysing percentage accuracy, correct item recall, inaccurate item recall, and confabulations individually. However, these measures share a common conceptual meaning and they contribute, both in combination and individually to understanding the efficacy of an episodic retrieval technique. Equally, the manipulations employed in this research are likely to affect eyewitness performance in more than one way, and hence need several criterion measures. As such performance measures have been considered in combination using multivariate analyses (MANOVA). Significant multivariate effects were further investigated by considering the univariate results (employing Bonferroni’s correction for multiple analyses). Significant findings were then examined using the Games Howell *post hoc* test.

### Overall Memorial Performance

Memorial performance, overall and as a function of retrieval phase are displayed in Table 2. A significant multivariate effect (combination of correct; inaccurate; confabulations) of retrieval support emerged, *F* (6, 92) = 9.841, *p*<.001, *η_p_* ^2^ = .039. Univariate analysis revealed that the multivariate effect emanated from the amount of correct and inaccurate information recalled, *F* (2, 48) = 31.679, *p*<.001, *η_p_* ^2^ = .39 and *F* (2, 48) = 3.413, *p* = .011, *η_p_* ^2^ = .31 respectively. Participants in the Sketch MRC condition recalled significantly more correct information, 95% CI [37.90, 43.28] than participants in both the MRC, *p* = .001, 95% CI [31.51, 36.25] and Control conditions, *p*<.001, 95% CI [24.13, 29.52]. Participants in the MRC condition recalled significantly more correct information than those in the Control, *p* = .010. Participants in the Sketch MRC recalled significantly fewer inaccurate items, 95% CI [2.42, 3.52] than those in the MRC, *p* = .008, 95% CI [3.69, 5.93] and Control, *p* = .004, 95% CI [3.29, 5.19] conditions with no significant difference between the latter two conditions. There were no differences across conditions for the amount of confabulated information recalled, *F* = 2.97, *p* = .061.

**Table 2:** Mean (SDs in parenthesis) overall and phase performance across conditions (N = 51).

	Retrieval Condition		
	Sketch MRC	MRC	Control
Total Correct Information	40.59 (5.25)	31.88 (4.06)	26.82 (5.28)
Free Recall Correct	31.47 (4.08)	24.44 (5.37)	22.12 (5.84)
Questioning Correct	9.12 (2.86)	7.45 (3.20)	4.71 (2.29)
Total InaccurateInformation	3.12 (1.36)	5.35 (1.32)	4.98 (1.52)
Free Recall Inaccurate	1.14 (0.84)	2.34 (0.67)	1.98 (0.70)
Questioning Inaccurate	1.99 (1.10)	2.98 (1.12)	3.01 (1.57)
Total Confabulations	1.62 (0.77)	1.65 (0.99)	1.79 (1.28)
Free Recall Confabulations	0.63 (0.49)	0.84 (0.69)	1.00 (0.95)
QuestioningConfabulations	1.00 (0.41)	0.81 (0.69)	0.79 (0.69)

### Interview Phase Performance

Interviews comprised two distinct recall attempts, namely a *free recall* (which included the MRC manipulation according to condition: No MRC; Sketch MRC; MRC) and *questioning* (see Table 2).

#### Free recall

Memorial performance in the Free Recall revealed a significant effect of interview for the amount of correct, *F* (2, 48) = 18.696, *p*<.001, *η_p_* ^2^ = .29, and confabulated information recalled, *F* (2, 48) = 5.302, *p* = .011, *η_p_* ^2^ = .15. Participants in the Sketch MRC recalled more correct information 95% CI [29.30, 33.60] than those in both the MRC, *p* = .009, 95% CI [22.00, 27.40] and Control conditions, *p*<.001, 95% CI [19.94, 24.30]. Participants in the MRC condition recalled significantly more correct information than those in the control, *p*<.001. Participants in the Sketch MRC confabulated less, 95% CI [−0.07, 0.42] than those in both the MRC, *p* = .03, 95% CI [0.34, 0.90] and Control conditions, *p* = .011, 95% CI [0.40, 0.90], with no significant difference between the latter two conditions. No significant difference emerged for the amount of inaccurate information recalled in the Free Recall phase, *F* = 3.000, *p* = .059.

#### Questioning phase

Analyses of participants’ performance in the questioning phase of interviews revealed a significant difference across conditions for the amount of correct information recalled, *F* (2, 48) = 11.267, *p*<.001, *η_p_* ^2^ = .31. Participants in the Sketch MRC recalled more correct information in the Questioning phase, 95% CI [7.81, 10.54] than in both the MRC, *p* = .030, 95% CI [5.35, 8.01] and Control conditions, *p*<.001, 95% CI [3.35, 6.06]. Participants in the Control condition recalled significantly fewer correct information items than those in the MRC condition, *p* = .010. No differences emerged for the amount of inaccurate or confabulated information, all *F*s <1.574, all *p*s >.060.

### Type of information recalled

The overall *type* of information recalled is displayed in Table 3 (Person; Object; Action). Across conditions, significant differences emerged in the numbers of correct person details, *F*(2, 47) = 13.746, *p*<.001, *η_p_* ^2^ = .31, and correct object details, *F*(2, 47) = 7.072, *p* = .002, *η_p_* ^2^ = .21. Participants in the Sketch MRC condition recalled more correct person details, 95% CI [21.00, 24.75] than participants in both the MRC, *p* = .001, 95% CI [15.42, 19.17], and Control conditions, *p*<.001, 95% CI 14.57, 18.43], with no significant difference between the latter conditions. Likewise, participants in the Sketch MRC recalled significantly more correct object details, 95% CI [7.68, 10.21], than participants in both the MRC, *p* = .006, 95% CI [4.94, 7.41], and Control conditions, *p* = .061, 95% CI [6.40, 7.95], with no significant difference between the latter conditions. No further significant differences emerged for type of information recalled, all *F*s <3.226, all *p*s >.05.

**Table 3:** Mean (SDs in parenthesis) type of information recalled across conditions (N = 51).

		Retrieval Condition		
		Sketch MRC	MRC	Control
Category	Subcategory			
ActionInformation	Correct	5.41 (1.72)	5.88 (1.49)	4.94 (1.43)
	Inaccurate	1.00 (0.86)	0.71 (0.77)	0.94 (0.90)
	Confabulations	0.24 (0.12)	0.65 (0.60)	0.52 (0.67)
PersonInformation	Correct	22.88 (4.47)	17.29 (3.27)	16.50 (3.69)
	Inaccurate	1.09 (0.98)	2.06 (1.19)	2.19 (1.10)
	Confabulations	0.94 (0.82)	0.82 (1.01)	1.63 (1.02)
ObjectInformation	Correct	8.94 (2.46)	6.18 (2.40)	6.18 (1.51)
	Inaccurate	1.06 (0.72)	1.05 (1.06)	1.29 (0.98)
	Confabulations	0.41 (0.60)	0.88 (0.78)	0.82 (0.89)

## Discussion

The purpose of this research was to investigate the efficacy of a novel environmental support tool for improving older adults’ episodic eyewitness performance. Specifically, it examined whether older witnesses might be better served by a Mental Reinstatement of Context (MRC) technique modified to include sketching when being interviewed about an experienced event. Theoretical accounts of aging guided the research, but in a departure from the typical laboratory mock witness paradigm the methodology was carefully adapted to mimic real life witnessing. In eyewitness situations, individuals frequently learn without intentional study, and are then required to consciously retrieve learned information. Incidental encoding does not allow rehearsal, and as such offers a more robust, and more realistic test of environmental support techniques for use in eyewitness settings. Accordingly, a live mock witness event was used, and face-to-face interviews were not conducted for forty-eight hours, thus bridging the gap between performance in artificial laboratory tasks and real world behaviour ([54], [55]). Moreover, this is the first older adult eyewitness research to have isolated the MRC component of the CI. Because the effectiveness of the MRC component with older adults has yet to be investigated individually, its contribution, or otherwise, is not well understood. This study goes part way toward filling this knowledge gap.

It was hypothesised that the Sketch MRC would improve memorial performance versus the current MRC and a no support Control. The findings support this prediction. Overall, the Sketch MRC outperformed the MRC and Control, eliciting over 22% and 29% more items of correct information, and reducing the amount of inaccurate recall by 44% and 37%, respectively, without a concomitant increase in the number of confabulations. It has long been argued that information within memory is organised hierarchically, and that specific episodic information is organised at a lower level than many other memories ([55], [56]). The Sketch MRC may stimulate a more rigorous search through the memory hierarchy in terms of implicitly encouraging more effortful generative retrieval attempts, rather than ‘allowing’ non-effortful direct retrieval that relies upon the spontaneous activation of episodic information. It is known that effortful processing at retrieval enhances recall performance [57], and that imaging improves episodic first response performance [58]. Both the standard and Sketch MRC encourage imaging. However, in the case of standard MRC participants are instructed to mentally image the encoding context, while in the sketch MRC participants are instructed to draw, which necessarily includes imaging [59]. Yet, the standard MRC was less effective across two significant performance measures (correct and inaccurate items).

Insight into the processes underpinning the Sketch MRC superiority effect is offered by considering the *nature* of episodic memory, and the *method* of recovering this type of information in an interview setting. Retrieving episodic information is a constructive process ([60], [61]), which in an eyewitness setting (in the UK and elsewhere) is necessarily directed and supported by an interviewer. Load theory proposes that increases in cognitive load (such as working memory load) deplete the resources available for attentional control and associated tasks, and that increased working memory and dual-task load also increases interference [62]. It is known that cognitive load is evoked by the instructions accompanying a task and also that goal-directed behaviour requires focusing attention on goal relevant stimuli. For instance, the ‘split-attention’ effect refers to the separate presentation of domain elements that demand simultaneous, internal processing [63], which is precisely what the standard MRC technique dictates. For older adults, who experience reduced processing efficiency and diminished working memory capacity ([36], [64], [38]), being asked to engage in the split attention, resource heavy MRC task (see Appendix S1), which does not allow externalisation, is likely to lessen the resources available for searching, retrieving, and verbalising episodic information, and so to reduce performance.

A further challenge associated with the MRC instructions concerns an integral focus on emotions experienced at the time of witnessing. When rememberers are instructed to focus on their emotions, rather than the to-be-remembered event itself, they make more errors in free recall ([65], [66]). The MRC includes instructions to ‘think about how you were feeling’; ‘think about what was going through your mind’, ‘think about who were you with that day’, ‘ think about what was happening around you’ etc. Conversely, the Sketch MRC does not. Rather, it allows participants to focus on those elements of the to-be-remembered event that are of individual import. As such output is pure (free of interviewer contamination) and rememberer-led, which may, albeit in part, account for differences in the *type* of information recalled in the Sketch MRC versus the standard MRC conditions. In the latter condition, fewer person and object details were recalled, which is concerning because this is investigatively important information. Incompatible retrieval cues are known to lessen recall performance, and the standard MRC technique employs cues that concern surroundings and the time leading up to the event (e.g., weather; presence of others; journey etc.) and internal (psychological) states. This may account for reduced person and object detail recall. In an eyewitness setting an interviewer has no option but to assist the rememberer to mentally reinstate the context by providing a set of non-suggestive, programmatic cues, presented similarly to every interviewee (the interviewer not having been present at the event, and having little idea as to what might constitute an effective retrieval cue). The benefits of the Sketch MRC may stem from the fact that participants are self-initiating, and as such are providing the most efficient and salient cues to further remembering ([67], [13]), although as yet it is unclear how this might affect recall of particular types of information. Future research should seek to investigate this.

What is clear from this study and the results of earlier work ([20], [21], [22]), is that the Sketch MRC offers an effective alternative to the MRC, and is worth adding to the toolbox of techniques already available to interviewers. However, this study is not without its limitations. The adult sample all lived independently in the community, but demographic information was not collected concerning levels of education and general health, all of which have the potential to affect memory performance. Future investigations should consider controlling for these variables. Finally, our discussion offers much fuel for future research in this area. It is right that theoretical accounts be applied to eyewitness memory settings in an attempt to understand the nature of real world behaviour. Given that most memory theory has its roots in laboratory word list experiments, contextualising theory in an applied setting presents significant methodological challenges. However, the integration of theory is critical to inform the development of theoretically-driven, empirically-based approaches and interventions.

To conclude, it was found that the Sketch MRC facilitated increased correct remembering in older adults, and reduced the number of inaccurate verbalisations, without a concomitant increase in confabulated intrusions. These findings are important because they illustrate the efficacy of appropriate environmental support at retrieval, using a paradigm that is absent from older adult eyewitness research, to date. Remembering often necessitates selecting goal relevant information in a competitive environment, where irrelevant and erroneous information may also be available. The Sketch MRC facilitates the selection of more correct goal relevant information during post event face-to-face interviews. To ensure that the justice system is fair, accessible, and delivers for all victims and witnesses [46], access must be widened to those in society, including older adults, who often present the greatest challenge. The results presented in this paper are a step toward this goal.

## Supporting Information

Appendix S1 **Mental Reinstatement of context instructions (Verbatim … indicates a 10 second pause).** “In a moment I am going to ask you to tell me what you remember about what happened last week. Before you begin I am going to ask you to try something that can often help people to remember more about what they have experienced. What I would like you to do is to close your eyes, or maybe look at a particular point in the room, and concentrate on the instructions I am going to give you. I would like you to listen silently to each of my instructions. I will pause between each instruction to give you time to do as I ask. To begin, I would like you to think back to the day that you came to the University … Think about what you had been doing that day … Think about how you were feeling …Who you were with that day … Who had you had spoken to … Think about getting ready to travel to the University … Think about how you travelled to the University… Picture in your mind your journey to the University … What was the weather like, try and get a good picture in your mind … Think about who you were with… Think back to when you arrived at the University … What could you smell … What could you hear… What could you see … Now picture in your mind the lecture theatre … Think about that room … Picture where you were sitting … Think about who you were sitting next to … How were you feeling … Think about what could you see … Think about that room … think about the windows … Think about the doors … When you have a really clear picture in your mind, please tell me everything that you remember …”.
(DOCX)

Appendix S2 **Sketch Mental Reinstatement of Context Instructions (verbatim). “**In a moment I am going to ask you to tell me what you remember about what happened last week. Before you begin I am going to ask you to try something that can often help people to remember more about what they have experienced. What I would like you to do is to draw about what happened Here are some pens and pencils and some paper You can draw what you want, just whatever reminds you about what happened When you are ready, you can start”.
(DOCX)
