# Supplementary material for: Drawing to Remember: External Support of Older Adults’ Eyewitness Performance
Source: PLoS One. 2013 Jul 29;8(7):e69937. doi: 10.1371/journal.pone.0069937 (PMC3726749; doi:10.1371/journal.pone.0069937)
Supplement: Appendix S1 — Mental Reinstatement of context instructions (Verbatim … indicates a 10 second pause). “In a moment I am going to ask you to tell me what you remember about what happened last week. Before you begin I am going to ask you to try something that can often help people to remember more about what they have experienced. What I would like you to do is to close your eyes, or maybe look at a particular point in the room, and concentrate on the instructions I am going to give you. I would like you to listen silently to each of my instructions. I will pause between each instruction to give you time to do as I ask. To begin, I would like you to think back to the day that you came to the University … Think about what you had been doing that day … Think about how you were feeling …Who you were with that day … Who had you had spoken to … Think about getting ready to travel to the University … Think about how you travelled to the University… Picture in your mind your journey to the University … What was the weather like, try and get a good picture in your mind … Think about who you were with… Think back to when you arrived at the University … What could you smell … What could you hear… What could you see … Now picture in your mind the lecture theatre … Think about that room … Picture where you were sitting … Think about who you were sitting next to … How were you feeling … Think about what could you see … Think about that room … think about the windows … Think about the doors … When you have a really clear picture in your mind, please tell me everything that you remember …”. (DOCX) [file pone.0069937.s001.docx]

Appendix A

**Mental Reinstatement of context instructions (Verbatim) … indicates a 10 second pause**

“ In a moment I am going to ask you to tell me what you remember about what happened last week. Before you begin I am going to ask you to try something that can often help people to remember more about what they have experienced. What I would like you to do is to close your eyes, or maybe look at a particular point in the room, and concentrate on the instructions I am going to give you. I would like you to listen silently to each of my instructions. I will pause between each instruction to give you time to do as I ask.

To begin, I would like you to think back to the day that you came to the University … Think about what you had been doing that day … Think about how you were feeling …W ho you were with that day … Who had you spoken to … Think about getting ready to travel to the University … Think about how you travelled to the University… Picture in your mind your journey to the University … What was the weather like, try and get a good picture in your mind … Think about who you were with. Think back to when you arrived at the University … What could you smell … What could you hear… What could you see … Now picture in your mind the lecture theatre … Think about that room … Picture where you were sitting … Think about who you were sitting next to … How were you feeling … Think about what could you see … Think about that room … think about the windows … Think about the doors ... When you have a really clear picture in your mind, please tell me … ”
