# Supplementary material for: Drawing to Remember: External Support of Older Adults’ Eyewitness Performance
Source: PLoS One. 2013 Jul 29;8(7):e69937. doi: 10.1371/journal.pone.0069937 (PMC3726749; doi:10.1371/journal.pone.0069937)
Supplement: Appendix S2 — Sketch Mental Reinstatement of Context Instructions (verbatim). “In a moment I am going to ask you to tell me what you remember about what happened last week. Before you begin I am going to ask you to try something that can often help people to remember more about what they have experienced. What I would like you to do is to draw about what happened Here are some pens and pencils and some paper You can draw what you want, just whatever reminds you about what happened When you are ready, you can start”. (DOCX) [file pone.0069937.s002.docx]

**Appendix B**

**Sketch Mental Reinstatement of Context Instructions (verbatim)**

In a moment I am going to ask you to tell me what you remember about what happened last week. Before you begin I am going to ask you to try something that can often help people to remember more about what they have experienced.

What I would like you to do is to draw about what happened

Here are some pens and pencils and some paper

You can draw what you want, just whatever reminds you about what happened

When you are ready, you can start
